# Bilateral Maxillary and Mandibular Periapical Abscesses

**DOI:** 10.7759/cureus.24434

**Published:** 2022-04-24

**Authors:** Cherian I Plamoottil, Muneet Gill, Rosa Flores, James Murray, Latha Ganti

**Affiliations:** 1 Emergency Medicine, HCA Florida UCF (University of Central Florida) Lake Nona Medical Center, Orlando, USA; 2 Emergency Medicine, Envision Physician Services, Plantation, USA; 3 Emergency Medicine, University of Central Florida College of Medicine, Orlando, USA; 4 Emergency Medicine, Brown University, Providence, USA; 5 Obstetrics and Gynecology, Lakeland Regional Health Medical Center, Lakeland, USA; 6 Emergency Medicine, HCA Florida Ocala Hospital, Ocala, USA

**Keywords:** dental abscess, facial edema, odontogenic infection, mandibular periapical abscess, maxillary periapical abscess

## Abstract

Odontogenic infections commonly arise from poor dental hygiene that forms dental caries, which can eventually progress to gingivitis and periodontitis. The authors present a case of facial swelling diagnosed as a periapical abscess with soft tissue swelling that extended into both the maxilla and mandible.

## Introduction

Facial infections of odontogenic origin are more common as people age. A 2005 study by the Centers for Disease Control noted that over 90% of adults have dental cavities as well as roughly 42% of children aged 6-19 years [1]. More than one in five people have signs of dental decay. Socioeconomic factors play a role in who is more likely to develop dental infections. In the age group of 20-64, dental caries are more commonly associated with those living under the poverty line. Additionally, in that same age group, non-Hispanic blacks are almost twice as likely to develop dental infections as non-Hispanic whites [2]. As dental infections begin to worsen, leading to more discomfort and pain, patients will eventually need dental procedures. Thirteen percent of adults will seek dental care for restorative procedures every four years. These restorative procedures include fillings, crowns, and bridges [3]. The effects of worsening dental health lead to an increased downstream cost not only from the procedures but also medications for pain control and antibiotics. 

Odontogenic disease can be classified on a spectrum where milder disease may present with minor discomfort and more severe disease can be much more dangerous and present with signs of systemic involvement. Minor disease includes reversible pulpitis that may present with severe toothache, lasting seconds, often provoked by temperature stimuli. As the disease progresses it becomes irreversible, leading to a longer duration of pain. If left untreated, the infection will spread to the gingiva and supporting structures of the tooth such as the alveolar bone. Patients with a periapical abscess will present with localized tooth pain and swelling. The disease can progress and will eventually invade the deeper structures of the neck. Externally, the patient will have signs of tooth decay as evidenced by black or yellow teeth if the patient is not edentulous. Patients may develop fevers, facial edema, as well as dysphagia or dysphonia, especially as facial edema worsens. Patients can be toxic appearing and have respiratory distress [4].

## Case presentation

**Figure 1:**
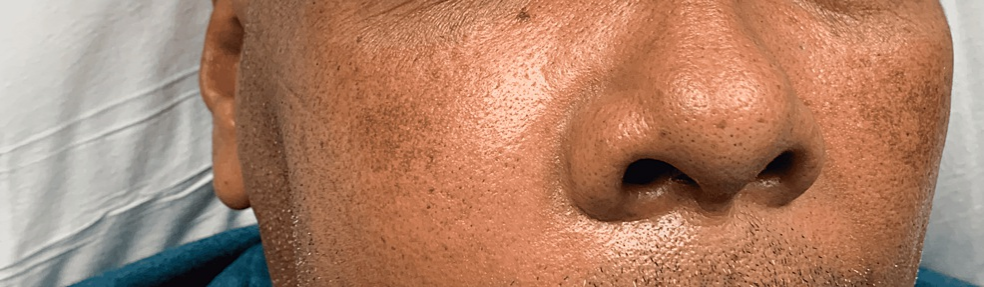
Clinical photograph of patient's facial swelling secondary to periapical abscess

A 54-year-old male, with a past medical history significant for diabetes and hypertension, presented to the emergency department for evaluation of right-sided facial swelling and pain. According to the patient, he noticed a right-sided pain in his upper jaw the day prior to presentation. In order to help with the pain and swelling, the patient took a nap with an icepack on his face. When he awoke he noticed that his right cheek was swollen to the size of a golf ball (Figure 1). The patient denied any fevers, chills, chest pain, or shortness of breath, and had an extensive smoking history.

**Figure 2:**
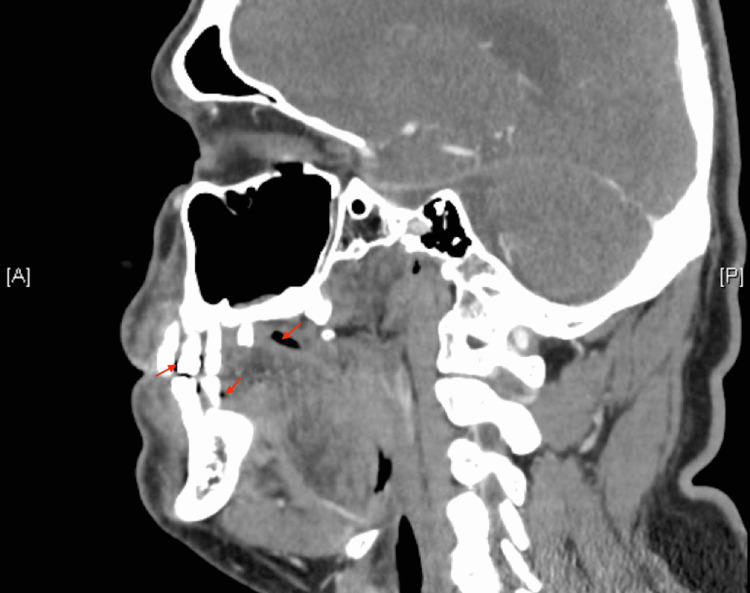
Computed tomography scan of facial bones revealing mandibular and maxillary periapical abscesses bilaterally with soft tissue thickening of the maxilla.

The patient’s oral exam revealed an atraumatic, patent airway with moist mucous membranes. There were no signs of a peritonsillar abscess or pooling of secretions. There was no malocclusion of the jaw or trismus. There was, however, visible decay of the first molar on the right. The neck was atraumatic and supple with no signs of meningismus. There was full range of motion to the neck with no notable swelling. The patient had basic labs drawn and also had a computed tomography scan of the facial bones. The scan revealed extensive dental caries with mandibular and maxillary periapical abscesses bilaterally (Figure 2).

There was also thickening of the maxilla anteriorly extending towards the right and caudad consistent with soft tissue infection. The patient received ampicillin-sulbactam and pain medications in the emergency department and had an appointment scheduled for the following morning with an oral surgeon to remove the infected teeth. He was instructed to continue taking clindamycin and over-the-counter analgesia for pain control. 

## Discussion

Dental infections are triggered by metabolic reactions that occur on biofilm overlying the teeth. This biofilm is more commonly referred to as plaque. These reactions lead to tooth decay and infection. With the underlying decay, bacteria will lead to enamel demineralization. Typically the mandibular third and second molar are the most commonly affected teeth [5]. As the enamel begins to deteriorate, bacteria will invade the teeth leading to pulpitis. Most commonly, dental infections are caused by resident oral flora, both anaerobic and aerobic. More common examples of aerobic bacteria involved with infection include *Streptococcus viridans* and *Staphylococcus aureus*. Anaerobic species include *Bacteroides* and *Prevotella* [6]. If pulpitis is not treated, the infection will spread to the bone causing periapical abscesses. An untreated periapical abscess will spread to adjacent osseous and deep neck structures leading to more systemic illnesses [7].

Treatment of these infections centers around the appropriate choice of antibiotics and, additionally, incision and drainage of the abscess or removal of the infected tooth. In addition, special care must be given to patients who have signs of airway compromise or deep space neck/fascial infections. Classically, penicillin has been the drug of choice as it covers a wide range of bacteria. Occasionally it can be paired with metronidazole to expand coverage. If a patient has a penicillin allergy, clindamycin is a suitable alternative. Immunocompromised patients should be covered with anti-pseudomonal antibiotics [5].

As stated earlier, untreated odontogenic infections can spread and cause devastating systemic illness. The most concerning complication of the infection is respiratory obstruction due to swelling of the airway. Constant vigilance is required to ensure that the patient’s airway remains secure. Other infections that may arise from a primary odontogenic infection include descending necrotizing mediastinitis, Lemierre’s syndrome, cervical necrotizing fasciitis, and brain and orbital abscesses. Should any of these complications arise, patients will likely need intravenous antibiotics and possible surgical interventions [8].

## Conclusions

Dental infections are very prevalent and can occur in nearly all age groups. Early and appropriate use of antibiotics and consultation with dentistry and oral maxillofacial surgical services can lead to optimal outcomes. Physicians should be aware of the more severe complications that arise from undertreated dental infections.

## References

[1] Beltrán-Aguilar ED, Barker LK, Canto MT (2005). Surveillance for dental caries, dental sealants, tooth retention, edentulism, and enamel fluorosis--United States, 1988-1994 and 1999-2002. MMWR Surveill Summ.

[2] Dye BA, Li X, Beltran-Aguilar ED (2012). Selected oral health indicators in the United States, 2005-2008. NCHS Data Brief.

[3] Erazo D, Whetstone DR (2021). Dental infections. StatPearls [Internet].

[4] Boykin MJ, Gilbert GH, Tilashalski KR, Shelton BJ (2003). Incidence of endodontic treatment: a 48-month prospective study. J Endod.

[5] Bahl R, Sandhu S, Singh K, Sahai N, Gupta M (2014). Odontogenic infections: microbiology and management. Contemp Clin Dent.

[6] Rega AJ, Aziz SR, Ziccardi VB (2006). Microbiology and antibiotic sensitivities of head and neck space infections of odontogenic origin. J Oral Maxillofac Surg.

[7] Laudenbach JM, Kumar SS (2020). Common dental and periodontal diseases. Dermatol Clin.

[8] Bali RK, Sharma P, Gaba S, Kaur A, Ghanghas P (2015). A review of complications of odontogenic infections. Natl J Maxillofac Surg.

